# The Symptoms of Lead Poisoning

**Published:** 1909-12-04

**Authors:** 


### SPECIAL ARTICLE.

### THE SYMPTOMS OF LEAD POISONING.

We do not think that enough experimental work has been done upon the question of how the symptoms of lead poisoning are produced. In most cases, even should the opportunity for post-mortem examination arise, the symptoms have been in existence for a long time, so that the initial changes in the tissues have passed away and only their after-effects remain. This is otherwise in the case of experiments carried out upon animals, in which the dosage can be so regulated that any required degree of lesion can be obtained, and the immediate post-mortem appearance ascertained. It will be interesting to see, therefore, whether the results described by Mr. Goadby and Dr. Goodbody are confirmed by others. They gave various salts of lead to animals, observed the symptoms during life, and recorded the effects as seen after death.

All the animals that died were submitted to an exhaustive necropsy. The post-mortem appearances were practically identical in all the animals: there was entire loss of subcutaneous and mesenteric fat, and the omentum was practically non-existent. Occasionally the diploë of the skull was deeply congested, more especially in the inoculated animals. The liver and intestines were injected, the former especially being engorged with blood. The lungs as a rule were normal, but rather full of blood, and in the animals subjected to inhalation the bronchial glands were found enlarged and often full of blood. There was practically no fat round the kidneys, but the capsules stripped easily. In one or two instances shrinkage of the cortex was noticed, but the most prominent symptom was the marked injection of the vessels on the surface of the kidney. The kidney on section was engorged with blood. No extravasation of blood was seen in the peritoneal cavity, except in one instance, where a hæmorrhage had occurred near the suprarenal capsules and in the region of the kidney. Small punctiform hæmorrhages were at times found in the stomach, in the duodenum, and the rest of the intestine; they were more marked in the lower end of the intestine, and particularly around the ileocæcal valve.

Further, in animals which had not received any lead by the mouth at all, and in which the poisoning was produced by the subcutaneous injection of a lead compound, there was very distinct blue-black staining of the upper half of the cæcum, extending up into the vermiform appendix. The spleen was normal. The muscles were normal but flabby; where paralysis had existed the corresponding muscle was found diminished in size. The surface of the brain appeared normal, but the vessels were somewhat larger than those of a normal animal. The vessels of the spinal cord were also somewhat increased in size, but there were no obvious hæmorrhages, nor were any macroscopical hæmorrhages found on making sections of the brain.

Portions of the heart, lungs, liver, spleen, intestines, stomach, and kidneys were preserved in formalin, cut in paraffin, and stained by Heidenhain's hæmatoxylin and eosin and by Van Giessen's method. Portions of the anterior crural nerve supplying the paralysed quadriceps extensor muscles were examined in celloidin, and also sections of the spinal cord and the cortex of the brain. This portion of the histology was undertaken by Dr. R. H. Clark. In all the tissues examined a distinct engorgement and increase in size of the blood-vessels were found; here and there the smaller vessels, particularly the capillaries, were over-distended, and in addition numbers of minute microscopical hæmorrhages were everywhere apparent in the brain, cord, liver, kidney, and lung. In the kidneys the same microscopical hæmorrhages were seen, with some slight changes in the epithelium of the tubules and a small amount of parenchymatous inflammation, no doubt due to the hæmorrhages which had taken place. The kidney, on the whole, seemed to suffer less than the lungs or liver. And in this connection it is worth noting that clinically no obvious signs of blood are noticeable in the urine of persons suffering from lead poisoning. Thickening of the arteries in the various organs was not well defined; the veins rather than the arteries appeared to be the source of the hæmorrhages. In the anterior crural nerves supplying the paralysed quadriceps extensor muscles minute hæmorrhages were found between the nerve bundles, but not generally invading the nerve fibres, nor was any degeneration found either in the nerves supplying the muscle or in the sections of the cord examined; in places it was seen that the hæmorrhage was producing pressure.

The whole histological picture was one of microscopic hæmorrhage occurring in the various organs and tissues, and it is evident that the minute hæmorrhages are the precursors of the fibroid and sclerotic changes that pathologists have seen in the tissues derived from cases of lead poisoning. Moreover, it is important to note in this connection that not a few of the symptoms in lead poisoning have been ascribed to stasis and vaso-motor spasm or vaso-constrictor spasm of the various blood vessels. In poisoning by nickel carbonyl Armit has shown that the lesions are due to the nickel and not to the CO, that the nickel is absorbed through the lung, and that injections of other nickel salts produce the same pathological changes as inhalation of nickel carbonyl. In animals poisoned by nickel hæmorrhages occur in all the tissues, especially the brain.

They conclude, therefore, from their experiments that the essential and primary action of lead intoxication is the production of minute and microscopical hæmorrhages in various portions of the body, including the nervous system; and that the clinical symptoms of lead palsy, and its good prognosis when treated early, are explainable by the presence of minute hæmorrhages in the peripheral nerves. The presence of these minute hæmorrhages in the nervous system also gives an explanation of the varied pathological findings of many previous workers.

